# SEAMHCRD deterministic compartmental model based on clinical stages of infection for COVID-19 pandemic in Sultanate of Oman

**DOI:** 10.1038/s41598-021-91114-5

**Published:** 2021-06-07

**Authors:** Abraham Varghese, Shajidmon Kolamban, Vinu Sherimon, Eduardo M. Lacap, Saad Salman Ahmed, Jagath Prasad Sreedhar, Hasina Al Harthi, Huda Salim Al Shuaily

**Affiliations:** 1 Department of Information Technology, Faculty of Mathematics, University of Technology and Applied Sciences, Muscat, Sultanate of Oman; 2 Department of Information Technology, Faculty of IT, University of Technology and Applied Sciences, Muscat, Sultanate of Oman; 3 Department of Information Technology, Faculty of Statistics, University of Technology and Applied Sciences, Muscat, Sultanate of Oman; 4 Head of Research, Royal Hospital, Muscat, Sultanate of Oman

**Keywords:** Mathematics and computing, Computational science

## Abstract

The present novel coronavirus (COVID-19) infection has engendered a worldwide crisis on an enormous scale within a very short period. The effective solution for this pandemic is to recognize the nature and spread of the disease so that appropriate policies can be framed. Mathematical modelling is always at the forefront to understand and provide an adequate description of the transmission of any disease. In this research work, we have formulated a deterministic compartmental model (SEAMHCRD) including various stages of infection, such as Mild, Moderate, Severe and Critical to study the spreading of COVID-19 and estimated the model parameters by fitting the model with the reported data of ongoing pandemic in Oman. The steady-state, stability and final pandemic size of the model has been proved mathematically. The various transmission as well as transition parameters are estimated during the period from June 4th to July 30th, 2020. Based on the currently estimated parameters, the pandemic size is also predicted for another 100 days. Sensitivity analysis is performed to identify the key model parameters, and the parameter gamma due to contact with the symptomatic moderately infected is found to be more significant in spreading the disease. Accordingly, the corresponding basic reproduction number has also been computed using the Next Generation Matrix (NGM) method. As the value of the basic reproduction number (R_0_) is 0.9761 during the period from June 4th to July 30th, 2020, the disease-free equilibrium is stable. Isolation and tracing the contact of infected individuals are recommended to control the spread of disease.

## Introduction

The novel coronavirus SARS-CoV-2 (COVID-19) is a spreadable disease that may be spread through contact and droplets^1–3^. The symptoms and signs of COVID-19 disease are visible after roughly 5.2 days (development period)^4^. The primary and common symptoms are fever, cough, fatigue, and sore throat, followed by other symptoms such as headache, diarrhea, the inability to sense taste and smell, muscle pain etc.^4,5^. In critical cases, infected people may develop bronchitis, pneumonia, severe acute respiratory distress syndrome (SARDS), multi-organ failure, and these may lead to death. In December 2019, the Wuhan city in China reported the first confirmed COVID-19 case; since then, the virus has spread universally. On March 11th, 2020, the World Health Organization (WHO) announced the COVID-19 epidemic as a pandemic^6^. The reported cases of COVID-19 have since been risen exponentially around the world reaching more than 200 nations^7^. As of September 11, 2020, the total number of infected cases is 28,316,605 and the total number of deaths is 913,284 all over the world^8^.

Nowadays mathematical modeling has been well recognized as an epidemiological tool to combat many infectious diseases. Many researchers attempted to study and model infectious diseases which is an interdisciplinary field where biological knowledge of epidemics is equally important along with the mathematical framework. The very first epidemiological mathematical model was traced back to the findings of Daniel Bernoulli in the eighteenth century to estimate the life expectancy in smallpox epidemics^9^. Ross constructed a set of mathematical equations to demonstrate the transmission of malaria parasites between mosquitoes and humans^10^. The Macdonald^11^ modified the work of Ross^10^ later and named it as well-known Ross-Macdonald models. The law of mass action was integrated into the Ross model by Kermack and McKendrick and new compartmental models were introduced^12^. These models later became the most extensively used basic structures usually called compartment models in infectious disease modeling. In the conventional compartmental model, the entire population is split into compartments according to the health status of people such as susceptible to being healthy individuals (S), infectious individuals (I), and recovered individuals (R) as in the SIR model^13^. The models’ SI, SIS, SIRS, SEIR (E-exposed but not infectious), SEIRS, MSIR (M-maternal immunity), MSEIR, and MSEIRS are other types of compartmental models that have been applied to many evolving communicable diseases like influenza and Ebola^14,15^.

Recently, Anastapoulou et al. proposed the SIR model for predicting COVID-19 outbreak^16^. Casella constructed a controlled SIR compartment model that includes the effect of delays and the outcome of different control strategies^17^. Giordano et al. developed a model called the SIDARTHE model that forecasts the effective control strategy of the COVID-19 pandemic situation in Italy^18^. It consists of eight classes such as susceptible (S), infected (I), diagnosed (D), ailing (A), recognized (R), threatened (T), healed (H) and extinct(E). The model differentiates between detected and non-detected among the infected cases and between life-threatening and non-life-threatening cases. Effect of lockdown, the effect of testing etc. were simulated. The research results established that social-distancing, an adequate number of tests and contact tracing will help to reduce the COVID-19 pandemic.

Researchers from Portugal and Spain proposed an ad-hoc compartmental model for COVID-19 in Wuhan^19^. In addition to categories such as susceptible, exposed, symptomatic, asymptomatic, and recovery, the research considered classes of individuals based on fatality, hospitalization, and super-spreaders. The basic reproduction number was computed to be 0.945, less than 1. So, the disease-free equilibrium was found to be stable. The sensitivity index of each of the parameters was calculated and the model was compared with the real data sets. The predicted model fits with the actual data of daily confirmed deaths.

The impact of social distancing and lockdown measures were studied in the research^20^ where a compartmental SEIR model was proposed. The research recommended that the total cases can be reduced by 90% if proper countermeasures are carried out. The dynamic behavior of the disease is studied in^21^ by developing a mathematical model by including isolation class. As per the research findings, the main cause of the disease spread is the contact between people. So, isolation of infected people is highly recommended to reduce the spread.

In this work, we suggest a new compartmental model that extends the classical SIR model by incorporating various infectious stage of the COVID-19 epidemic in the Sultanate of Oman for 145 days. As of September 11, 2020, out of 88,337 confirmed cases in the Sultanate of Oman, 4250 are active, 83,325 have recovered, and 762 are dead^8^.

## Materials and methods

In this research, we formulate the transmission mechanism of COVID-19 using a compartmental model which is deterministic in nature. We have collected patient wise demographic data including symptoms from Royal hospital, Oman after taking formal ethical approval. The data highlighted the number of patients admitted in ICU, admitted in the hospital, and not required admission in the hospital. Their duration of stay in the hospital (1 to 30 days) is also given in the data depending on the severity of the disease. Also, we have studied various symptoms, along with the severity of the diseases which leads to arrive at different compartments. We have made a detailed study on the various stages from mild to severe that COVID-19 patient is undergoing while they are under treatment. These studies prompted us to make a compartment model that incorporates mild, moderate, critical, and severe cases separately instead of taking all together as an infected case.

To formulate the mathematical model, the total population is grouped into eight mutually exclusive compartments based on their disease status. The class of Susceptible individuals (S) are those who have never been infected with and thus have no immunity against COVID-19. Susceptible individuals become exposed once they are infected with the disease. The Exposed individuals (E) are those who are in the infected stage, but not yet infectious to others. The class (A) of infected individuals are those with no symptom or mild symptom developed. These individuals may have symptoms like fever and cough but with normal chest X-ray result. These individuals may either improve or progress to the Moderate stage of the disease. The Moderate class (M) of individuals suffers from a moderate infection symptom like fever and cough and may even have mild pneumonia but do not need hospitalization. In this stage, they are likely to show symptoms of fever and cough and with chest X-ray result that indicate major bilateral abnormality, pneumonia, or infiltrations/patchy shadowing. These individuals may either improve or progress to the severe stage of the disease.

The hospitalized individuals (H) suffer from a severe infection and have severe pneumonia and need hospitalization. These people show indications of serious pneumonia that prompts dyspnea, blood oxygen saturation that is less than or equal to 93%, respiratory frequency which is more than or equivalent to 30 breaths/min at rest, the ratio of the partial pressure of arterial oxygen to fraction of inspired oxygen being less than or equal to 300 mmHg, and/or lung infiltration that is more than 50% within 24 to 48 hours. Such cases require immediate hospitalization and supplemental oxygen. These individuals may either recover or progress to the critical stage of the disease. The class of critical (C) individuals are affected by a critical infection like a failure in the respiratory system, septic shock, and/or dysfunction in multiple organs and require treatment in an Intensive care unit (ICU), often with the requirement of mechanical ventilation. These individuals may either recover or die suffering from the disease during the time. The Recovered individuals (R) are those who have recovered and are expected to be immune to future infection with COVID-19. The class of individuals (D) are those who have died because of COVID-19. Depending on the severity of diseases like mild, moderate, severe, and critical, the Infected people are sent to appropriate COVID care centers or at home isolation. The authorities are monitoring their progress daily and taking appropriate actions^22^.

## Model and equations

**Figure 1:**
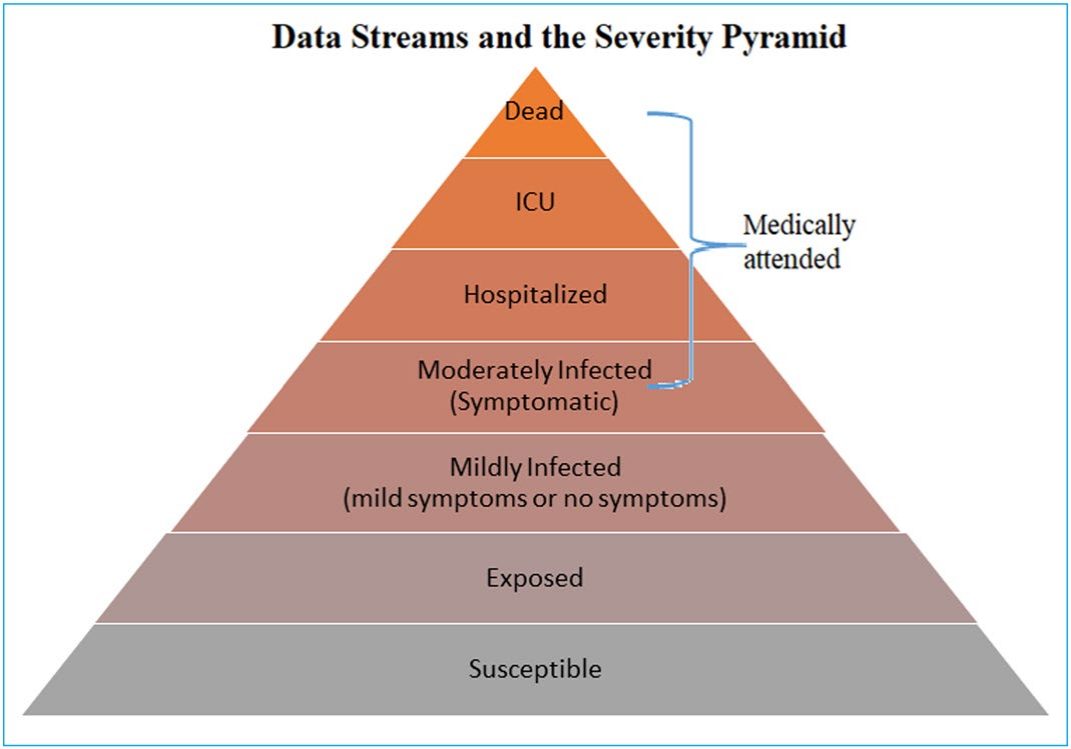
The different stages of the compartment model.

Figure 1 depicts the various stages of the proposed compartment model.

**Figure 2:**
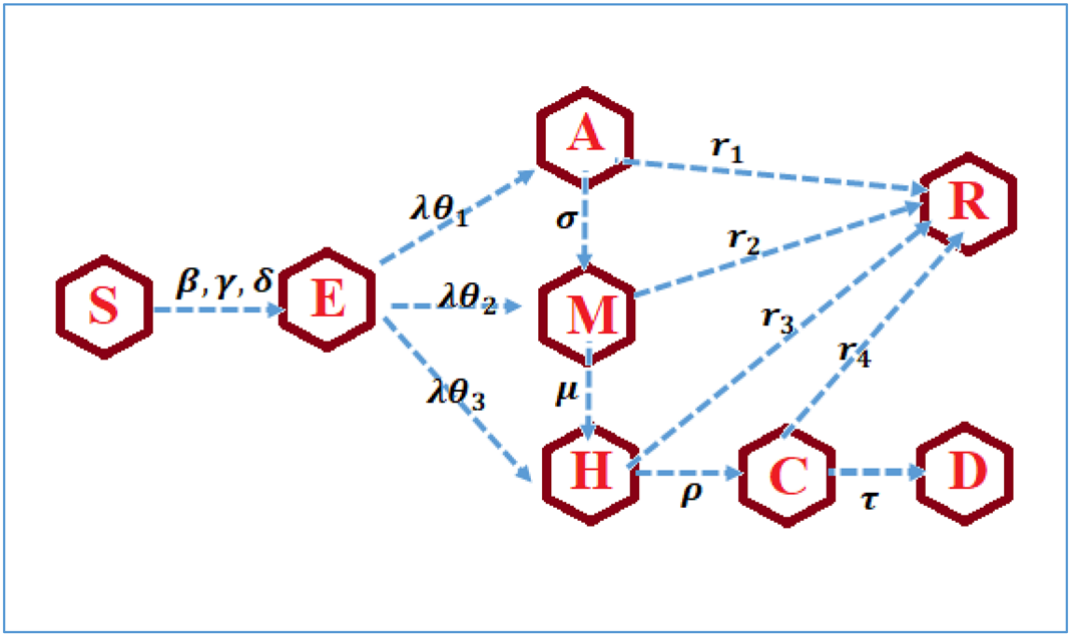
The overview of the model.

At any time, t, total population N (t) = S (t) + E (t) + A(t) + M (t) + H(t) + C (t) + R(t) + D(t). These 8 compartments are governed by 8 dynamic system of non-linear differential equations that vary with time, and nature of the solution and the stability of the system is investigated. Figure 2 depicts the diagram of the overview of the model. The corresponding equations and the description of the various parameters involved are given in Eq. (1). The compartment C is completely isolated to strict preventive measures and proper care in ICU. Hence the transmission from C is considered as negligibly small.

$$S^{\prime}(t) = - S(t)\left[ {\beta A(t) + \gamma M(t) + \delta H(t)} \right]$$

$$E^{\prime}(t) = S(t)\left[ {\beta A(t) + \gamma M(t) + \delta H(t)} \right] - \lambda (\theta_{1} + \theta_{2} + \theta_{3} )E(t)$$

$$A^{\prime}(t) = \lambda \theta_{1} E(t) - \left( {\sigma + r_{1} } \right)A(t)$$

1 $$M^{\prime}(t) = \lambda \theta_{2} E(t) + \sigma A(t) - \left( {\mu + r_{2} } \right)M(t)$$

$$H^{\prime}(t) = \lambda \theta_{3} E(t) + \mu M(t) - \left( {\rho + r_{3} } \right)H(t)$$

$$C^{\prime}(t) = \rho H(t) - (\tau + r_{4} )C(t)$$

$$R^{\prime}(t) = r_{1} A(t) + r_{2} M(t) + r_{3} H(t) + r_{4} C(t)$$

$$D^{\prime}(t) = \tau C(t)$$

We choose $$\theta_{3} = 1 - (\theta_{1} + \theta_{2} )$$.

Table 1 provides the description the parameter.

**Table 1:** Description of the parameter.

$\alpha$	Transmission rate due to contact with asymptomatic exposed individuals	Transmission parameters
$\beta$	Transmission rate due to contact with asymptomatic/mildly infected individuals	
$\gamma$	Transmission rate due to contact with the symptomatic moderately infected	
$\delta$	Transmission rate due to the contact with the symptomatic severely infected	
$\theta_{1}$	Detection rate for the asymptomatic/mildly infected	Transition parameters
$\theta_{2}$	Detection rate for the symptomatic moderately infected	
$\theta_{3}$	Detection rate for the symptomatic severely infected	
$\sigma$	Asymptomatic/mildly infected become symptomatic moderately infected	
$\mu$	Symptomatic moderately infected become symptomatic severely infected	
$\rho$	Symptomatic severely infected become symptomatic critically infected	
$\tau$	Mortality rate for the critically infected	
$\lambda$	Exit rate from E	
$\varepsilon$	Exit rate from $E_{N}$	
$\upsilon$	Exit rate from $E_{T}$	
$r_{1}$	Recovery rate for the asymptomatic/mild infected	
$r_{2}$	Recovery rate for the symptomatic moderate infected	
$r_{3}$	Recovery rate for the symptomatic severe infected	
$r_{4}$	Recovery rate for critical infection	

### Analysis of the model

#### Nonnegativity of solutions

The model (1) represents a bilinear system consists of 8 differential equations. The system is considered positive when all the compartmental variables assume non-negative values for $t \ge 0$. $R(t)$ and $D(t)$ are cumulative variables.

##### **Proposition 1**

Consider a system F(t) = (S, E, A, M, H, C, R, D) with the initial conditions $F(0) \ge 0$, then the solutions of (1) are non-negative for all $t \ge 0$.

##### Proof

The integrating factor of first equation in (1) is $e^{{\int\limits_{0}^{t} {\left( {\beta A(\phi ) + \gamma M(\phi ) + \delta H(\phi } \right))d\phi } }}$.

So, the solution is $S(t){\kern 1pt} {\kern 1pt} e^{{\int\limits_{0}^{t} {\left( {\beta A(\phi ) + \gamma M(\phi ) + \delta H(\phi } \right))d\phi } }} = S(0)$

$$S(t){\kern 1pt} = S(0){\kern 1pt} {\kern 1pt} {\kern 1pt} \left( {e^{{ - \int\limits_{0}^{t} {\left( {\beta A(\phi ) + \gamma M(\phi ) + \delta H(\phi } \right))d\phi } }} } \right) \ge 0$$

Similarly, we can establish other variables also. Thus $F\left( t \right) \ge 0$ for all $t \ge 0$.

The compartmental system satisfies mass conservative property, and it can be verified easily from (1) that $S^{\prime}(t) + E^{\prime}(t) + A^{\prime}(t) + M^{\prime}(t) + H^{\prime}(t) + C^{\prime}(t) + R^{\prime}(t) + D^{\prime}(t) = 0$. Hence the total population is a constant. When we consider each variable as the fraction of population, we can assume $S(t) + E(t) + A(t) + M(t) + H(t) + C(t) + R(t) + D(t) = 1$, where 1 represents the total population.

Assuming an initial condition $S(0)$, $E(0)$, $A(0)$,$M(0)$, $H(0)$, $C(0)$, $R(0)$, $D(0)$ summing to 1, it is possible to prove that the variables converge to an equilibrium $\overline{S} \ge 0$, $\overline{E} = 0$, $\overline{A} = 0$,$\overline{M} = 0$, $\overline{H} = 0$, $\overline{C} = 0$, $\overline{R} \ge 0$, $\overline{D} \ge 0$ with $\overline{S} + \overline{R} + \overline{D} = 1$. So only the susceptible, recovered and dead populations are eventually present. This means that the epidemic phenomenon has come to an end. All the possible equilibria are given by $(\overline{S},0,0,0,0,0,\overline{R},\overline{D})$ with $\overline{S} + \overline{R} + \overline{D} = 1$. In our model (1) the rate of the transmission parameter values $\beta ,\gamma$ is to be reduced by strict social measures. Also, the rate of the main transition parameters $\sigma$,$\mu$ depends on the strictness of the home isolation and quarantine process. Even if the strict procedure is adopted in hospital admission, there is always a chance for the disease to spread from the hospital. This is because long-time exposure to huge numbers of infected patients directly increments the risk of infection among healthcare workers. In addition to this, inadequate personal protection of healthcare workers, shortage of personal protective equipment (PPE), as well as lack of organized training, practice, professional administration, and guidance also may aid the spread the disease. The increase in awareness of personal protection, availability of sufficient personal protection equipment (PPE), and proper awareness would play a significant role in reducing the risk of infection among healthcare workers^23^. Hence by considering all these factors, we incorporated compartment class H in our model in determining the spread of the disease, specifically in calculating the basic reproduction number.

The model has mainly three subsystems: The first subsystem is the susceptible individuals (S), the second subsystem is the infected and exposed individuals ($E$, $A$, $M$, $H$, $C$) which are non-zero in the transmission state , the third subsystem includes the recovered and removed individuals ($R$,$D$). The second subsystem is more important in the epidemic study. When this subsystem is zero the remaining variables are at equilibrium. The variables $R$, $D$, are monotonically increasing and converge to the values $\overline{R}$, $\overline{D}$, respectively and $S$ is monotonically decreasing and converges to $\overline{S}$ if and only if the second subsystem converges to zero.

#### Basic reproduction number (R_0_)

It is not possible to predict the endemicity of the disease based on the increase or decrease in the number of cases alone. We need to find equilibria and linearization about each equilibrium to determine whether the disease is pandemic or not. If an equilibrium is unstable with all susceptible population, then it is an epidemic, and if it is asymptotically stable, then it is the end of the pandemic. To get a meaningful realization of the situation, the basic reproduction number (R_0_) is calculated, and this determines whether there is an epidemic or not. The basic reproduction number (R_0_) is debatably the most significant quantity in infectious disease epidemiology. It is among the quantities most critically evaluated for evolving infectious diseases in epidemic states, and its value delivers insight when scheduling control interventions for conventional infections. It can be found easily whether the disease-free equilibrium of the system exists and is calculated from this system of the equation after converting to next-generation matrix (NGM).

If R_0_ < 1, the infection stops, and if R_0_ > 1, there is an epidemic. The basic reproduction number (R_0_) is characterized as the number of secondary diseases brought about by a single infected individual from a susceptible population and is an indicator of the severity of the disease. Diekmann et al.^1^ and Van den Driessche and Watmough^2^ proposed a generalized approach to determine the basic reproduction number using the next generation matrix approach. The linearization of (1) the disease-free equilibrium is of the form $(\overline{S},0,0,0,0,0,\overline{R},\overline{D})$. The infectious sub-system from (1) can be written as

$$E^{\prime}(t) = S(t)\left[ {\beta A(t) + \gamma M(t) + \delta H(t)} \right] - \lambda (\theta_{1} + \theta_{2} + \theta_{3} )E(t)$$

$$A^{\prime}(t) = \lambda \theta_{1} E(t) - \left( {\sigma + r_{1} } \right)A(t)$$

2 $$M^{\prime}(t) = \lambda \theta_{2} E(t) + \sigma A(t) - \left( {\mu + r_{2} } \right)M(t)$$

$$H^{\prime}(t) = \lambda \theta_{3} E(t) + \mu M(t) - \left( {\rho + r_{3} } \right)H(t)$$

The Jacobian matrix from (2) is

$$J_{I} = \left[ {\begin{array}{*{20}c} { - p_{1} } & \beta & \gamma & \delta \\ {{\lambda}\theta_{1} } & { - p_{2} } & 0 & 0 \\ {{\lambda}\theta_{2} } & \sigma & { - p_{3} } & 0 \\ {{\lambda}\theta_{3} } & 0 & \mu & { - p_{4} } \\ \end{array} } \right]$$

where $$p_{1} = \lambda \left( {\theta_{1} + \theta_{2} + \theta_{3} } \right) = \lambda$$, $$p_{2} = \sigma + r_{1}$$, $$p_{3} = \mu + r_{2}$$, $$p_{4} = \rho + r_{3}$$,

Now the Jacobian matrix is decomposed into two matrices (transmission matrix and Transition matrix).

$$T = \left[ {\begin{array}{*{20}c} 0 & \beta & \gamma & \delta \\ 0 & 0 & 0 & 0 \\ 0 & 0 & 0 & 0 \\ 0 & 0 & 0 & 0 \\ \end{array} } \right]$$ $$\Sigma = \left[ {\begin{array}{*{20}c} { - p_{1} } & 0 & 0 & 0 \\ {\lambda \theta_{1} } & { - p_{2} } & 0 & 0 \\ {\lambda \theta_{2} } & \sigma & { - p_{3} } & 0 \\ {\lambda \theta_{3} } & 0 & \mu & { - p_{4} } \\ \end{array} } \right]$$ The NGM with classical domain is denoted by D_C_. Here, we assume an auxiliary matrix E of K is of the form: $$K = \left[ {\begin{array}{*{20}c} 1 & 0 & 0 & 0 \\ 0 & 0 & 0 & 0 \\ 0 & 0 & 0 & 0 \\ 0 & 0 & 0 & 0 \\ \end{array} } \right]$$.

Now.

$$D_{C} = - K^{\prime}T\Sigma^{ - 1} K = \left[ {\begin{array}{*{20}c} {\frac{{\beta \theta_{1} }}{{p_{2} }} + \frac{{\gamma \sigma \theta_{1} }}{{p_{2} p_{3} }} + \frac{{\gamma \theta_{2} }}{{p_{3} }} + \frac{{\delta \sigma \mu \theta_{1} }}{{p_{2} p_{3} p_{4} }} + \frac{{\delta \mu \theta_{2} }}{{p_{3} p_{4} }} + \frac{{\delta \theta_{3} }}{{p_{4} }}} & 0 & 0 & 0 \\ 0 & 0 & 0 & 0 \\ 0 & 0 & 0 & 0 \\ 0 & 0 & 0 & 0 \\ \end{array} } \right]$$.

From this, the basic reproduction number by considering the classical domain matrix is the spectral radius of the NGM.

3 $$R_{0} = trace(D_{C} ) = \frac{{\beta \theta_{1} }}{{p_{2} }} + \frac{{\gamma \theta_{2} }}{{p_{3} }} + \frac{{\delta \theta_{3} }}{{p_{4} }} + \frac{{\gamma \sigma \theta_{1} }}{{p_{2} p_{3} }} + \frac{{\delta \mu \theta_{2} }}{{p_{3} p_{4} }} + \frac{{\delta \sigma \mu \theta_{1} }}{{p_{2} p_{3} p_{4} }}$$

#### Steady state analysis

##### **Proposition 2**

The disease-free equilibrium is unstable for $R_{0} > 1$ , and stable for $R_{0} < 1$.

##### Proof

The dynamical matrix of the linearized system around the equilibrium $(\overline{S},0,0,0,0,0,\overline{R},\overline{D})$ is.

4 $$J = \left[ {\begin{array}{*{20}c} 0 & 0 & { - \beta \overline{S}} & { - \gamma \overline{S}} & { - \delta \overline{S}} & 0 & 0 & 0 \\ 0 & {p_{1} } & {\beta \overline{S}} & {\gamma \overline{S}} & {\delta \overline{S}} & 0 & 0 & 0 \\ 0 & {\lambda \theta_{1} } & { - p_{2} } & 0 & 0 & 0 & 0 & 0 \\ 0 & {\lambda \theta_{2} } & \sigma & { - p_{3} } & 0 & 0 & 0 & 0 \\ 0 & {\lambda \theta_{3} } & 0 & \mu & { - p_{4} } & 0 & 0 & 0 \\ 0 & 0 & 0 & 0 & \rho & { - p_{5} } & 0 & 0 \\ 0 & 0 & {r_{1} } & {r_{2} } & {r_{3} } & {r_{4} } & 0 & 0 \\ 0 & 0 & 0 & 0 & 0 & \tau & 0 & 0 \\ \end{array} } \right]$$

The matrix (4) has three null eigenvalues and five eigenvalues which are the roots of the equation.

$$\begin{aligned} P(s) & \equiv \frac{{\beta \lambda \theta_{1} }}{{\left( {s + p_{1} } \right)\left( {s + p_{2} } \right)}} + \frac{{\gamma \sigma \lambda \theta_{1} }}{{\left( {s + p_{1} } \right)\left( {s + p_{2} } \right)\left( {s + p_{3} } \right)}} + \frac{{\gamma \lambda \theta_{2} }}{{\left( {s + p_{1} } \right)\left( {s + p_{3} } \right)}} \\ & \quad + \frac{{\delta \sigma \mu \lambda \theta_{1} }}{{(s + p_{1} )(s + p_{2} )(s + p_{3} )(s + p_{4} )}} + \frac{{\delta \mu \lambda \theta_{2} }}{{(s + p_{1} )(s + p_{3} )(s + p_{4} )}} + \frac{{\delta \lambda \theta_{3} }}{{(s + p_{1} )(s + p_{4} )}} - 1 = 0 \\ \end{aligned}$$

Then $$P(0) = R_{0} - 1$$, because $$p_{1} = \lambda \left( {\theta_{1} + \theta_{2} + \theta_{3} } \right) = \lambda$$.

Now consider the following cases

##### **Case 1**

Suppose $R_{0} > 1$. Then $P(0) > 0$. Also $P(s) \to - 1$ as $s \to \infty$. Since $P(s)$ is a continuous function of $s$, hence by Bolzano theorem on continuous function implies that $P(s_{i} ) = 0$ for some $s_{i} > 0$, at least one eigenvalue must be positive. So, in this case, the equilibrium is unstable.

##### **Case II**

Suppose $$R_{0} < 1$$. Then $$P(0) < 0$$.

Assume that $P(s) = 0$ has a root of the form $x + iy$ where $x,y \in R$ and $x \ge 0$. Then $P(x + iy) = 0$.

Now

$$\begin{aligned} \left| {P(x + iy) + 1} \right| & \le \frac{{\beta \lambda \theta_{1} }}{{\left| {\left( {s + p_{1} } \right)} \right|\left| {\left( {s + p_{2} } \right)} \right|}} + \frac{{\gamma \sigma \lambda \theta_{1} }}{{\left| {\left( {s + p_{1} } \right)} \right|\left| {\left( {s + p_{2} } \right)} \right|\left| {\left( {s + p_{3} } \right)} \right|}} + \frac{{\gamma \lambda \theta_{2} }}{{\left| {\left( {s + p_{1} } \right)} \right|\left| {\left( {s + p_{3} } \right)} \right|}} \\ & \quad + \frac{{\delta \sigma \mu \lambda \theta_{1} }}{{\left| {(s + p_{1} )} \right|\left| {(s + p_{2} )} \right|\left| {(s + p_{3} )} \right|\left| {(s + p_{4} )} \right|}} + \frac{{\delta \mu \lambda \theta_{2} }}{{\left| {(s + p_{1} )} \right|\left| {(s + p_{3} )} \right|\left| {(s + p_{4} )} \right|}} + \frac{{\delta \lambda \theta_{3} }}{{\left| {(s + p_{1} )} \right|\left| {(s + p_{4} )} \right|}} \\ & \quad \le \frac{{\beta \lambda \theta_{1} }}{{\left( {x + p_{1} } \right)\left( {x + p_{2} } \right)}} + \frac{{\gamma \sigma \lambda \theta_{1} }}{{\left( {x + p_{1} } \right)\left( {x + p_{2} } \right)\left( {x + p_{3} } \right)}} + \frac{{\gamma \lambda \theta_{2} }}{{\left( {x + p_{1} } \right)\left( {x + p_{3} } \right)}} \\ & \quad + \frac{{\delta \sigma \mu \lambda \theta_{1} }}{{(x + p_{1} )(x + p_{2} )(x + p_{3} )(x + p_{4} )}} + \frac{{\delta \mu \lambda \theta_{2} }}{{(x + p_{1} )(x + p_{3} )(x + p_{4} )}} + \frac{{\delta \lambda \theta_{3} }}{{(x + p_{1} )(x + p_{4} )}} \\ & \quad \le \frac{{\beta \lambda \theta_{1} }}{{p_{1} p_{2} }} + \frac{{\gamma \sigma \lambda \theta_{1} }}{{p_{1} p_{2} p_{3} }} + \frac{{\gamma \lambda \theta_{2} }}{{p_{1} p_{3} }} + \frac{{\delta \sigma \mu \lambda \theta_{1} }}{{p_{1} p_{2} p_{3} p_{4} }} + \frac{{\delta \mu \lambda \theta_{2} }}{{p_{1} p_{3} p_{4} }} + \frac{{\delta \lambda \theta_{3} }}{{p_{1} p_{4} }} = R_{0} < 1 \\ \end{aligned}$$

This shows $1 < 1$, clearly a contradiction. Hence all roots of the equation $P(s) = 0$ have the form $x + iy$ where $x,y \in R$ and $x < 0$. Thus, all eigenvalues of the coefficient matrix have negative real part and this case equilibrium is stable, the solution of (1) die out exponentially^9^.

#### Final pandemic size relations

##### **Proposition 3**

The limit values $\overline{S} = \mathop {\lim }\limits_{t \to \infty } S(t)$ , $\overline{R} = \mathop {\lim }\limits_{t \to \infty } R(t)$ and $\overline{D} = \mathop {\lim }\limits_{t \to \infty } D(t)$ or positive initial conditions, are given by.

5 $$l_{0} + R_{0} \left( {S(0) - \overline{S}} \right) = \log \left( {\frac{S(0)}{{\overline{S}}}} \right)$$

6 $$\overline{R} = R(0) + l_{R} + R_{R} \left( {S(0) - \overline{S}} \right)$$

7 $$\overline{D} = D(0) + f_{D} + R_{D} \left( {S(0) - \overline{S}} \right)$$

where $$l_{0} = - a^{T} B^{ - 1} x(0)$$ , $$l_{R} = - b^{T} B^{ - 1} x(0)$$, $$R_{R} = - b^{T} B^{ - 1} D$$, $$l_{D} = - c^{T} B^{ - 1} x(0)$$ and $$R_{D} = - c^{T} B^{ - 1} D$$.

##### Proof

Define $x = [E,A,M,H,C]^{T}$, we can write the subsystem as.

8 $$x^{\prime}(t) = B{\kern 1pt} x(t) + D{\kern 1pt} u(t) = \left[ {\begin{array}{*{20}c} { - p_{1} } & 0 & 0 & 0 & 0 \\ {\lambda \theta_{1} } & { - p_{2} } & 0 & 0 & 0 \\ {\lambda \theta_{2} } & \sigma & { - p_{3} } & 0 & 0 \\ {\lambda \theta_{3} } & 0 & \mu & { - p_{4} } & 0 \\ 0 & 0 & 0 & \rho & { - p_{5} } \\ \end{array} } \right]x(t) + \left[ {\begin{array}{*{20}c} 1 \\ 0 \\ 0 \\ 0 \\ 0 \\ 0 \\ \end{array} } \right]u(t)$$

9 $$y_{S} (t) = a^{T} x(t) = \left[ {\begin{array}{*{20}c} 0 & \beta & \gamma & \delta & 0 \\ \end{array} } \right]x(t)$$

10 $$y_{R} (t) = b^{T} x(t) = \left[ {\begin{array}{*{20}c} 0 & {r_{1} } & {r_{2} } & {r_{3} } & {r_{4} } \\ \end{array} } \right]x(t)$$

11 $$y_{D} (t) = c^{T} x(t) = \left[ {\begin{array}{*{20}c} 0 & 0 & 0 & 0 & \tau \\ \end{array} } \right]x(t)$$

12 $$u(t) = S(t)y_{S} (t)$$

where $$p_{1} = \lambda (\theta_{1} + \theta_{2} + \theta_{3} )$$, $$p_{2} = \sigma + r_{1}$$, $$p_{3} = \mu + r_{2}$$, $$p_{4} = \rho + r_{3}$$, $$p_{5} = \tau + r_{4}$$.

and the other variables in the differential Eq. (1) can be written as

13 $$S^{\prime}(t) = - S(t){\kern 1pt} {\kern 1pt} y_{S} (t)$$

14 $$R^{\prime}(t) = {\kern 1pt} {\kern 1pt} y_{R} (t)$$

15 $$D^{\prime}(t) = {\kern 1pt} y_{D} (t)$$

The number of infectives always approaches zero and the number of susceptible always approaches a positive limit as $t \to \infty$.

From (8)

16 $$\begin{gathered} S^{\prime}(t) = - S(t){\kern 1pt} {\kern 1pt} y_{S} (t) \hfill \\ - \frac{S^{\prime}(t)}{{S(t){\kern 1pt} }} = {\kern 1pt} y_{S} (t) \Rightarrow - \int\limits_{0}^{\infty } {\frac{S^{\prime}(t)}{{S(t){\kern 1pt} }}} = \int\limits_{0}^{\infty } {{\kern 1pt} y_{S} (t)} \hfill \\ \Rightarrow \int\limits_{0}^{\infty } {{\kern 1pt} y_{S} (t)} = \log \left( {\frac{S(0)}{{\overline{S}}}} \right) \hfill \\ \end{gathered}$$

From (7)

$$\begin{gathered} \int\limits_{0}^{\infty } {x^{\prime}(\phi )d\phi } = B\int\limits_{0}^{\infty } {{\kern 1pt} x(\phi )} d\phi + D\int\limits_{0}^{\infty } {{\kern 1pt} u(\phi )d\phi } \hfill \\ x(\infty ) - x(0) = B\int\limits_{0}^{\infty } {{\kern 1pt} x(\phi )} d\phi + D\int\limits_{0}^{\infty } {{\kern 1pt} S(\phi )y_{S} (\phi )d\phi } \hfill \\ \end{gathered}$$

from (12).

Since $$x(\infty ) = 0$$

$$- x(0) = B\int\limits_{0}^{\infty } {{\kern 1pt} x(\phi )} d\phi - D\int\limits_{0}^{\infty } {{\kern 1pt} S^{\prime}(\phi )d\phi }$$

from (13)

17 $$- x(0) = B\int\limits_{0}^{\infty } {{\kern 1pt} x(\phi )} d\phi - D\left( {\overline{S} - S(0)} \right)$$

Pre multiply by $a^{T} B^{ - 1}$ and using (9) we have

$$- a^{T} B^{ - 1} x(0) = a^{T} B^{ - 1} B\int\limits_{0}^{\infty } {{\kern 1pt} x(\phi )} d\phi - a^{T} B^{ - 1} D\left( {\overline{S} - S(0)} \right)$$

$$- a^{T} B^{ - 1} x(0) = \int\limits_{0}^{\infty } {{\kern 1pt} y_{S} (\phi )} d\phi - a^{T} B^{ - 1} D\left( {\overline{S} - S(0)} \right)$$

$$l_{0} = \log \left( {\frac{S(0)}{{\overline{S}}}} \right) + R_{0} \left( {\overline{S} - S(0)} \right)$$

from (16)

where $l_{0} = - a^{T} B^{ - 1} x(0)$ and it is easy to show that $R_{0} = - a^{T} B^{ - 1} D$. So

$$l_{0} + R_{0} \left( {S(0) - \overline{S}} \right) = \log \left( {\frac{S(0)}{{\overline{S}}}} \right)$$

The expressions (6) and (7) can be proved easily by pre-multiplying (17) by $b^{T} B^{ - 1}$ and $c^{T} B^{ - 1}$ respectively.

Note that with an initial condition $[E(0) > 0,A(0) = M(0) = H(0) = C(0) = 0]$,we can compute $l_{0}$ as $l_{0} = R_{0} E(0)$ and for long term prediction it has to be adjusted by considering $l_{0} = - a^{T} B^{ - 1} x(t_{0} )$ where B includes new parameter values. In (5) also, it is adjusted accordingly by $S(t_{0} )$.

## Extension of the model by including exposed period

The study on COVID-19 reveals the fact that transmission of disease happens in various stages, namely symptomatic transmission, pre-symptomatic transmission, and asymptomatic transmission. In the case of a symptomatic transmission, the infected persons develop signs and symptoms compatible with COVID-19 virus infection and transfers to the contacted person(s). Specifically, symptomatic transmission denotes the transmission from a person while they are suffering from the symptoms and this transmission takes place through respiratory droplets or by contact with the contaminated objects and surfaces^5,30–34^. Research studies on virus infection reveal that flaking of COVID-19 virus is peak in the upper respiratory tract (nose and throat) early during the disease, which is, within the first three days from the start of symptoms^24–26^. Preliminary data proposes that people may be more infectious around the time of the beginning of the symptoms as compared to the later stages of the disease^35–37^.

In the case of pre-symptomatic transmission, there is an incubation period (on average 5–6 days and can be extended up to 14 days) which is the duration between the first day of infection and symptom onset, is, however, can be up to 14 days. During this period, some infected persons can be contagious. Therefore, transmission from a pre-symptomatic case can occur up to a certain extent before symptom onset. Various studies^24–29^ reveal that people can be tested positive for COVID-19 from 1 to 3 days before the development of symptoms. Thus, it is probable that infected people could transmit the disease before the onset of any visible symptoms. It is very significant to understand that pre-symptomatic transmission still needs the virus to be spread through infectious droplets or through touching contaminated surfaces.

**Figure 3:**
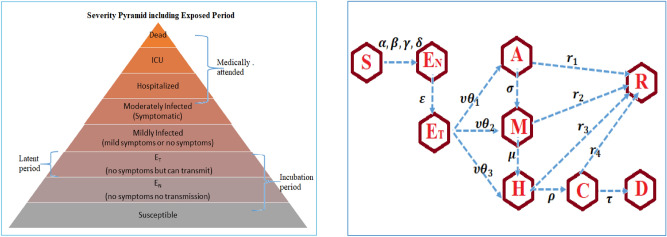
Extension of the model by including exposed period.

While in asymptomatic transmission, a person is infected with COVID-19, but no visible symptoms. Asymptomatic transmission refers to the transmission of the infection from an infected individual who does not show any symptoms. Even though there is no solid report on asymptomatic transmission, we cannot avoid the possibility that it may occur. In some countries, asymptomatic cases have been reported through an effective contact tracing mechanism. WHO frequently monitors all evolving evidence about this and is delivering updates whenever more information becomes available. So in our model (1), the exposed classes are divided into two separate classes namely $E_{N}$ defines no symptom or transmission and $E_{T}$ with no symptom but can transmit the virus (“pre-symptomatic transmission”), based on the various studies^24–29^ about COVID-19 pre-symptomatic infection. The rate of exit from $E_{N}$ is $\varepsilon$ and from $E_{T}$ is $\upsilon$. Accordingly, the modified diagram by expanding exposed case is given in Fig. 3. The modified equation of (1) is also given in (18).

$$S^{\prime}(t) = - S(t)\left[ {\alpha E_{T} + \beta A(t) + \gamma M(t) + \delta H(t)} \right]$$

$$E_{N} ^{\prime}(t) = S(t)\left[ {\alpha E_{T} + \beta A(t) + \gamma M(t) + \delta H(t)} \right] - \varepsilon E_{N} (t)$$

$$E_{T} ^{\prime}(t) = \varepsilon E_{N} (t) - \upsilon (\theta_{1} + \theta_{2} + \theta_{3} )E_{T} (t)$$

$$A^{\prime}(t) = \upsilon \theta_{1} E{}_{T}(t) - \left( {\sigma + r_{1} } \right)A(t)$$

18 $$M^{\prime}(t) = \upsilon \theta_{2} E_{T} (t) + \sigma A(t) - \left( {\mu + r_{2} } \right)M(t)$$

$$H^{\prime}(t) = \upsilon \theta_{3} E_{T} (t) + \mu M(t) - \left( {\rho + r_{3} } \right)H(t)$$

$$C^{\prime}(t) = \rho H(t) - (\tau + r_{4} )C(t)$$

$$R^{\prime}(t) = r_{1} A(t) + r_{2} M(t) + r_{3} H(t) + r_{4} C(t)$$

$$D^{\prime}(t) = \tau C(t)$$

We choose $$\theta_{3} = 1 - (\theta_{1} + \theta_{2} )$$.

Accordingly, the basic reproduction number using next generation matrix can be written as

19 $$R_{0} = \frac{\alpha }{{p_{1} }} + \frac{{\beta \theta_{1} }}{{p_{2} }} + \frac{{\gamma \theta_{2} }}{{p_{3} }} + \frac{{\delta \theta_{3} }}{{p_{4} }} + \frac{{\gamma \sigma \theta_{1} }}{{p_{2} p_{3} }} + \frac{{\delta \mu \theta_{2} }}{{p_{3} p_{4} }} + \frac{{\delta \sigma \mu \theta_{1} }}{{p_{2} p_{3} p_{4} }}$$

where $$p_{1} = \upsilon (\theta_{1} + \theta_{2} + \theta_{3} ) = \upsilon$$, $$p_{2} = \sigma + r_{1}$$, $$p_{3} = \mu + r_{2}$$, $$p_{4} = \rho + r_{3}$$, $$p_{5} = \tau + r_{4}$$.

The model including exposed period was implemented in MATLAB and findings are depicted in “Results” section.

## Results

The simulation of the extended model is implemented in MATLAB. The data for this study has been taken from the official Tarassud Plus application, Ministry of Health, Oman^38^ and the sources^38,39^. The data of Oman during the period June 4th, 2020 to July 30th, 2020 (about 57 days) has been used for this study. During this period, the country has undergone lockdown, partial lockdown and relaxation. The given data is categorized into 9 compartments (S, $E_{N}$, $E_{T}$,, A, M, H, C, R, D) as mentioned in the methodology Section 2.1. The data for the study is given in Table 2.

**Table 2:** Oman Dataset.

Date	S	E_N_	E_T_	A	M	H	C	R	D	AC
04 June 2020	5,087,015	3779	1512	2103	8411	226	58	3451	67	10,798
05 June 2020	5,085,870	4047	1619	2255	9021	227	60	3451	72	11,563
06 June 2020	5,084,484	4373	1749	2435	9740	251	67	3451	72	12,493
07 June 2020	5,083,195	4675	1870	2598	10,393	290	75	3451	75	13,356
08 June 2020	5,082,466	4764	1906	2651	10,603	283	75	3793	81	13,612
09 June 2020	5,081,582	4887	1955	2717	10,870	291	85	4152	83	13,963
10 June 2020	5,080,643	5066	2026	2819	11,277	293	85	4329	84	14,474
11 June 2020	5,080,179	4635	1854	2568	10,274	308	92	6623	89	13,242
12 June 2020	5,078,943	4720	1888	2617	10,468	309	92	7489	96	13,486
13 June 2020	5,077,465	5057	2023	2808	11,231	315	94	7530	99	14,448
14 June 2020	5,075,829	5223	2089	2902	11,608	313	100	8454	104	14,923
15 June 2020	5,074,805	5209	2084	2892	11,570	317	104	9533	108	14,883
**16 June 2020**	**5,074,461**	**4923**	**1969**	**2727**	**10,907**	**327**	**105**	**11,089**	**114**	**14,066**
17 June 2020	5,073,602	4958	1983	2740	10,962	362	102	11,797	116	14,166
18 June 2020	5,073,221	4702	1881	2591	10,363	378	103	13,264	119	13,435
19 June 2020	5,072,302	4750	1900	2616	10,464	389	102	13,974	125	13,571
20 June 2020	5,071,364	4780	1912	2630	10,518	411	99	14,780	128	13,658
21 June 2020	5,070,395	4826	1930	2658	10,633	396	101	15,552	131	13,788
22 June 2020	5,068,426	5086	2034	2805	11,219	407	100	16,408	137	14,531
23 June 2020	5,066,891	5241	2096	2891	11,566	421	97	17,279	140	14,975
24 June 2020	5,065,529	5398	2159	2981	11,924	417	100	17,972	142	15,422
25 June 2020	5,063,764	5683	2273	3143	12,571	417	107	18,520	144	16,238
26 June 2020	5,062,552	5740	2296	3174	12,694	426	105	19,482	153	16,399
27 June 2020	5,061,618	5751	2300	3177	12,710	431	113	20,363	159	16,431
**28 June 2020**	**5,060,247**	**5875**	**2350**	**3249**	**12,997**	**423**	**118**	**21,200**	**163**	**16,787**
29 June 2020	5,059,492	5764	2306	3186	12,745	423	115	22,422	169	16,469
30 June 2020	5,058,482	5764	2306	3183	12,732	437	117	23,425	176	16,469
01 July 2020	5,057,174	5896	2358	3260	13,041	426	120	24,162	185	16,847
02 July 2020	5,055,713	5967	2387	3303	13,212	420	114	25,318	188	17,049
03 July 2020	5,054,086	6148	2459	3401	13,606	447	113	26,169	193	17,567
04 July 2020	5,052,728	6277	2511	3473	13,894	452	116	26,968	203	17,935
05 July 2020	5,051,600	6317	2527	3494	13,975	458	121	27,917	213	18,048
06 July 2020	5,049,885	6430	2572	3555	14,222	465	129	29,146	218	18,371
07 July 2020	5,048,916	6221	2488	3436	13,746	464	127	31,000	224	17,773
08 July 2020	5,047,610	6289	2516	3474	13,898	469	128	32,005	233	17,969
09 July 2020	5,045,847	6464	2586	3575	14,298	466	129	33,021	236	18,468
**10 July 2020**	**5,043,627**	**6701**	**2680**	**3703**	**14,811**	**501**	**130**	**34,225**	**244**	**19,145**
11 July 2020	5,042,520	6718	2687	3709	14,835	517	133	35,255	248	19,194
12 July 2020	5,040,974	6881	2752	3798	15,194	525	143	36,098	257	19,660
13 July 2020	5,038,318	7232	2893	4000	15,998	519	146	37,257	259	20,663
14 July 2020	5,036,613	7458	2983	4129	16,516	514	149	37,987	273	21,308
15 July 2020	5,034,630	7675	3070	4252	17,007	530	139	39,038	281	21,928
16 July 2020	5,033,173	7768	3107	4299	17,197	549	149	40,090	290	22,194
17 July 2020	5,031,431	7856	3142	4347	17,386	555	157	41,450	298	22,445
18 July 2020	5,030,131	7848	3139	4337	17,349	574	164	42,772	308	22,424
19 July 2020	5,029,014	7819	3128	4318	17,271	585	165	44,004	318	22,339
**20 July 2020**	**5,026,990**	**8023**	**3209**	**4436**	**17,744**	**574**	**170**	**45,150**	**326**	**22,924**
21 July 2020	5,025,493	8030	3212	4438	17,753	582	169	46,608	337	22,942
22 July 2020	5,023,669	8147	3259	4507	18,027	577	165	47,922	349	23,276
23 July 2020	5,023,714	7330	2932	4037	16,147	589	169	51,349	355	20,942
24 July 2020	5,022,822	7149	2860	3937	15,750	568	170	53,007	359	20,425
25 July 2020	5,021,755	7149	2860	3938	15,751	570	167	54,061	371	20,426
26 July 2020	5,020,659	7113	2845	3922	15,688	545	167	55,299	384	20,322
27 July 2020	5,019,942	6873	2749	3782	15,126	552	177	57,028	393	19,637
28 July 2020	5,019,450	6620	2648	3641	14,565	528	181	58,587	402	18,915
29 July 2020	5,019,274	6271	2508	3442	13,769	522	184	60,240	412	17,917
30 July 2020	5,018,978	6061	2424	3324	13,295	511	187	61,421	421	17,317

We investigated the model parameters and corresponding effective reproduction number R_0_ during the period from June 4th to July 30th when the country has undergone various intervened measures to control the disease.

### Parameter estimation

Mathematical models are useful in capturing patterns in data, thereby predicting the nature of a pandemic to take the right policy to control it. Hence it is very essential to find out the best-fit parameters that give the closest correspondence between model predictions and data. The parameter estimation is done based on parameterized nonlinear functions which solve the $\min \left( {\sum {\left\| {F(x_{i} - y_{i} } \right\|^{2} } } \right),$ where $F(x_{i} )$ is a nonlinear function and ${y}_{i}$ is data. The parameters for each period are chosen in such a way that it gives the minimum value for R_0_.

**Figure 4:**
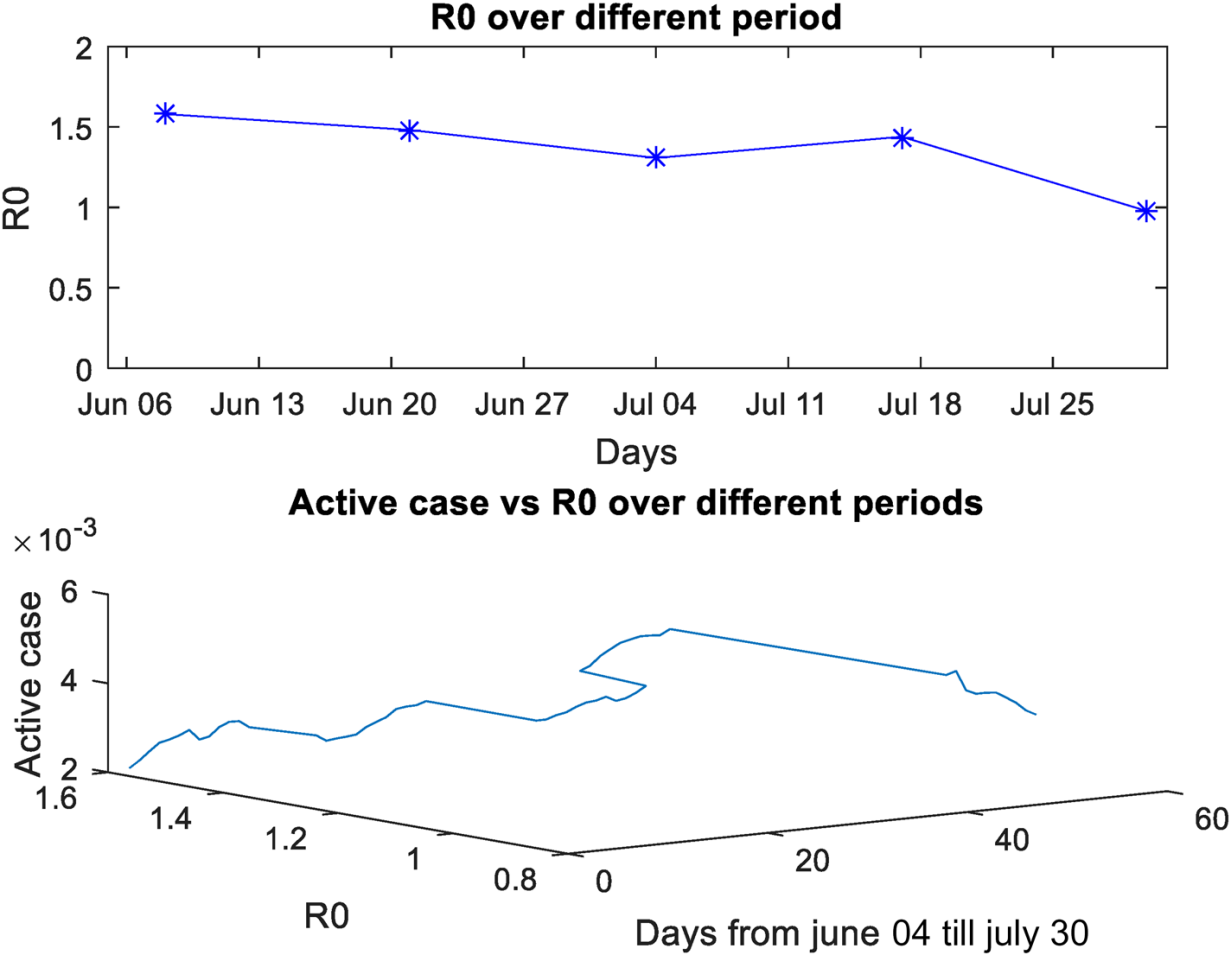
R_0_ and active case versus time.

**Figure 5:**
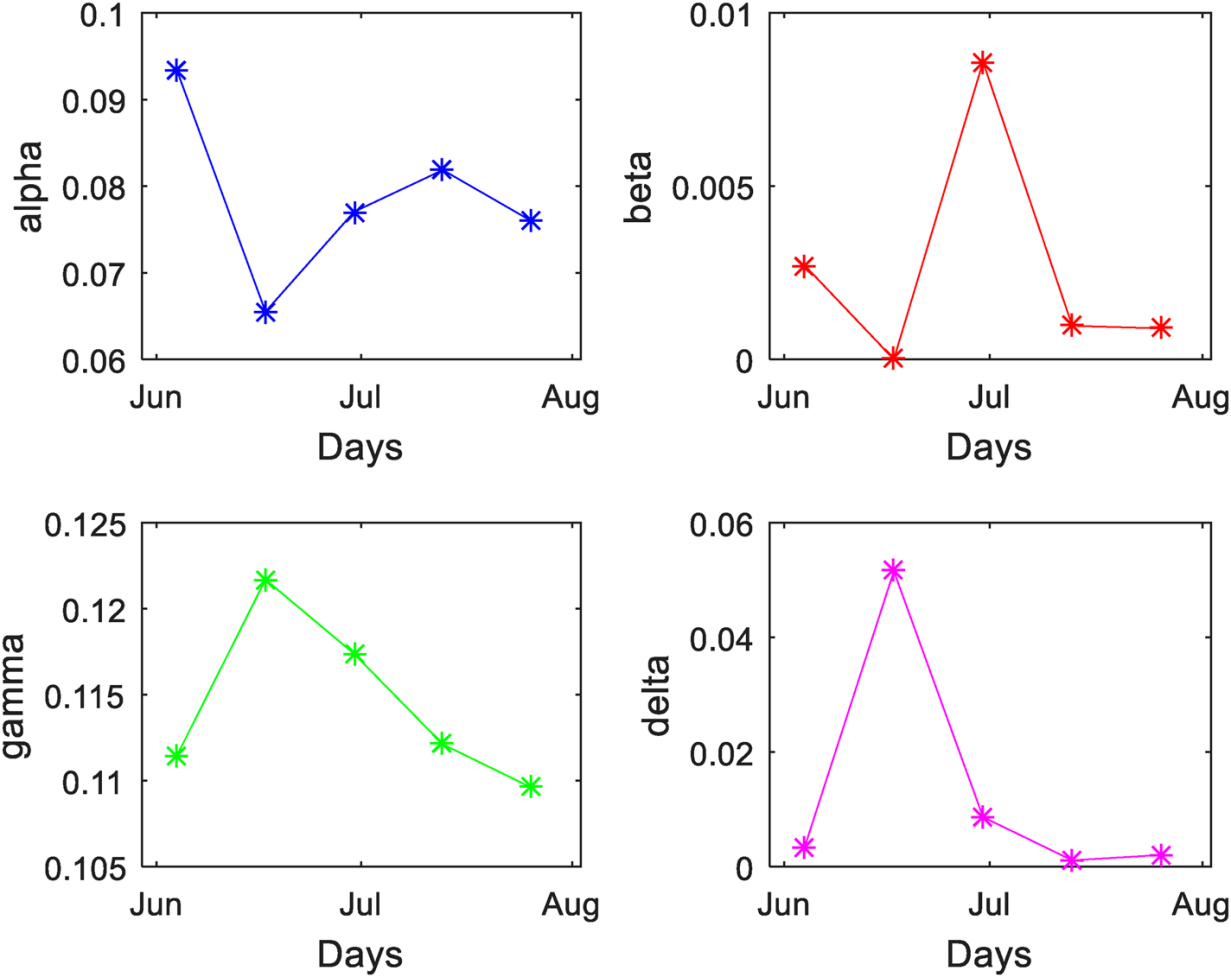
The transmission parameters versus time.

Figure 4 shows the R_0_ over a different period, and active cases versus R_0_. Figure 5 shows the various transmission parameters over a period from June to August based on the fitted data’.

- **Period 1 (4th June—15th June 2020)**
  The estimated parameters from the model are, α = 0.0934, β = 0.0027, γ = 0.1115, δ = 0.0034, σ = 0.0439, µ =0.0363, ρ = 0.0249, τ = 0.0100, r_1_ = 0.0745, r_2_ = 0.0364, r_3_ = 0.0281, r_4_ = 0.0227, θ_1_ = 0.4032, θ_2_ = 0.9101, θ_3_ = 0.0352. The corresponding reproduction number is R_0_ = 1.5802.
- **Period 2 (16th June—27th June 2020)**
  The estimated parameters from the model in the given period are: α = 0.0655, β = 3.12e-06, γ = 0.1217, δ = 0.0515, σ = 0.10449, µ = 0.0516, ρ = 0.0267, τ = 0.00999, r_1_ = 0.04296, r_2_ = 0.0809, r_3_ = 0.02769, r_4_ = 0.0155,_1_ = 0.313, θ_2_ = 0.855, θ_3_ = 0.0126. The corresponding reproduction number is R_0_ = 1.4811.
- **Period 3(28th June—10th July 2020)**
  The estimated parameters are from the model are: α = 0.0770, β = 0.00858, γ = 0.11738, δ = 0.00866, σ = 0.0805, µ = 0.0276, ρ = 0.0260, τ = 0.00304, r_1_ = 0.04538, r_2_ = 0.0689, r_3_ = 0.02557, r_4_ = 02.53e-05, θ_1_ = 0.2984, θ_2_ = 0.739, θ_3_ = 0. 000341. The corresponding reproduction number is Rv = 1.3071.
- **Period 4 (11th July—20th July 2020)**
  During the period, the resulting parameter value estimates are: α = 0.0818, β = 0.000969, γ = 0.1121, δ = 0.00112, σ = 0.00952, µ = 0.0109, ρ = 0.02679, τ = 0.00999, r_1_ =0.0591, r_2_ = 0.05591, r_3_ = 0.02823, r_4_ = 0.00089, θ_1_ = 0.2278, θ_2_ = 0.7479, θ_3_ = 9.59e-05. The corresponding R_0_ is 1.4392.
- **Period 5 (21st July—30th July 2020)**
  At the end of this period, the estimates are α = 0.07597, β = 0.000893, γ = 0.10960, δ = 0.00205, σ = 0.03988, µ = 0.01678, ρ = 0.02577, τ = 0.00999, r_1_ =0.0455, r_2_ = 0.09789, r_3_ = 0.02673, r_4_ = 2.571e-08, θ_1_ = 0.2661, θ_2_ = 0.7753, θ_3_ = 0.01952.The corresponding R_0_ is 0.9761.

### Prediction of pandemic size

**Figure 6:**
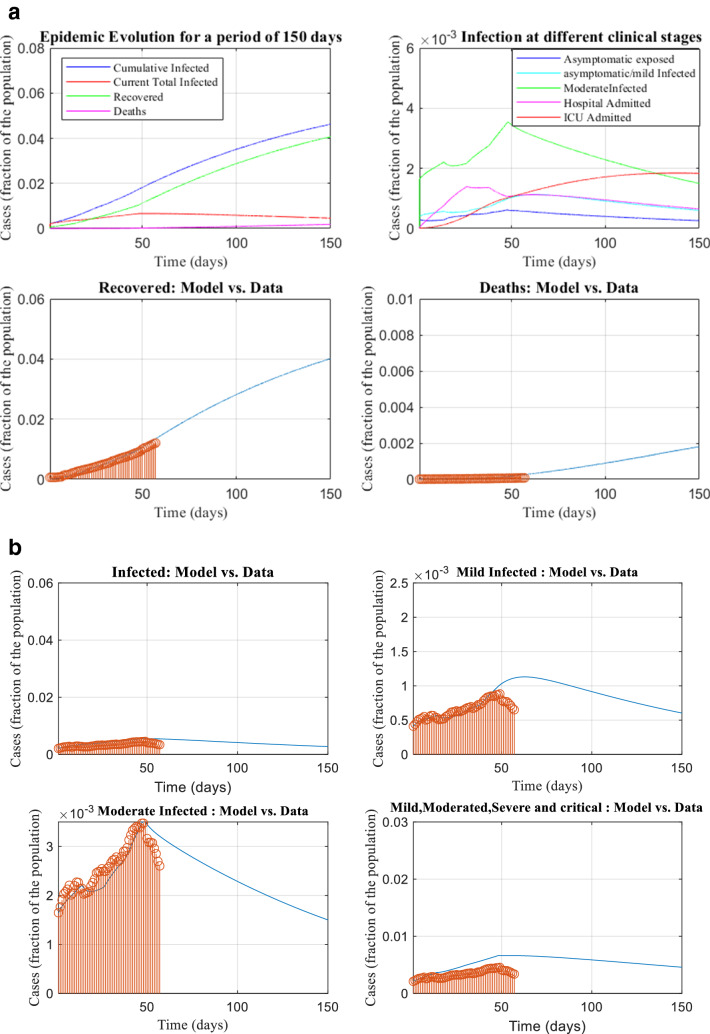
(**a**) The pandemic size of different clinical stage versus time—infected case, the recovered case and death case. (**b**) The pandemic size of different clinical stage versus time—mild, moderate and all the cases together.

The data from June 4th to July 30th (57 days) is fitted using 9 differential equations as given in (1.18) and the corresponding parameters are estimated. Figure 6a depicts the projection of infected case, recovered case and deaths, and Fig. 6b depicts that of mild, moderate and altogether.

**Figure 7:**
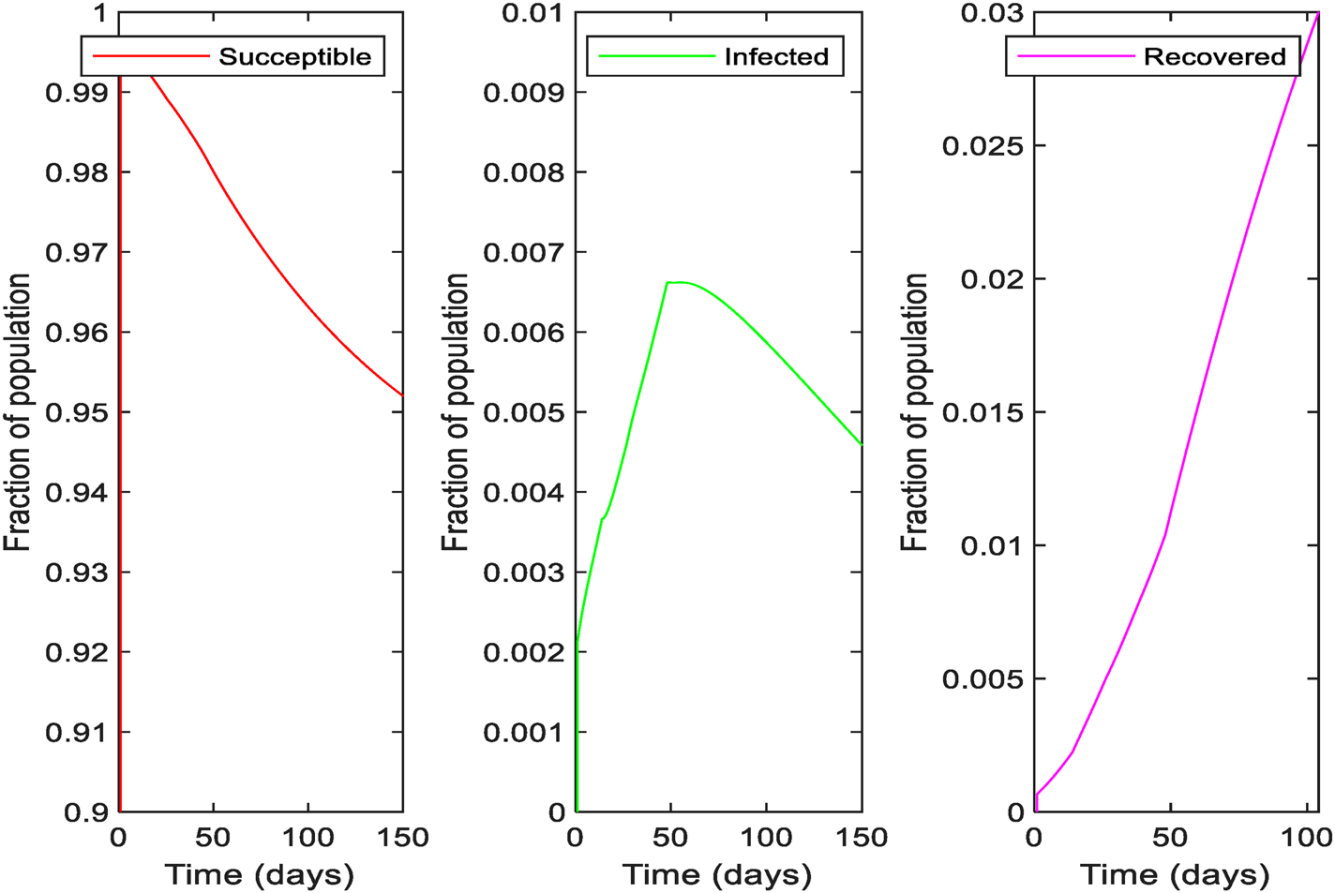
The prediction of susceptible, recovered cases and cases of deaths.

Based on the parameters obtained during **Period 5 (21st July–30th July 2020),** with the assumption that the policy remains the same, we predicted the susceptible, infected and recovered cases and these are presented in Fig. 7. It is observed that even after 150 days from June 4, 95% of the total population will remain susceptible (i.e.,not get infected) and 0.4% are infected provided we can keep basic reproduction number ${R}_{0}$ less than 1 by ensuring social distancing.

### Sensitivity analysis of the model with respect to varying parameters

We analyzed the sensitivity of the model on infected and, recovered cases and cases of death cases concerning transmission parameters, which would help the policymakers to plan appropriate strategies to mitigate the spread of the disease.

**Figure 8:**
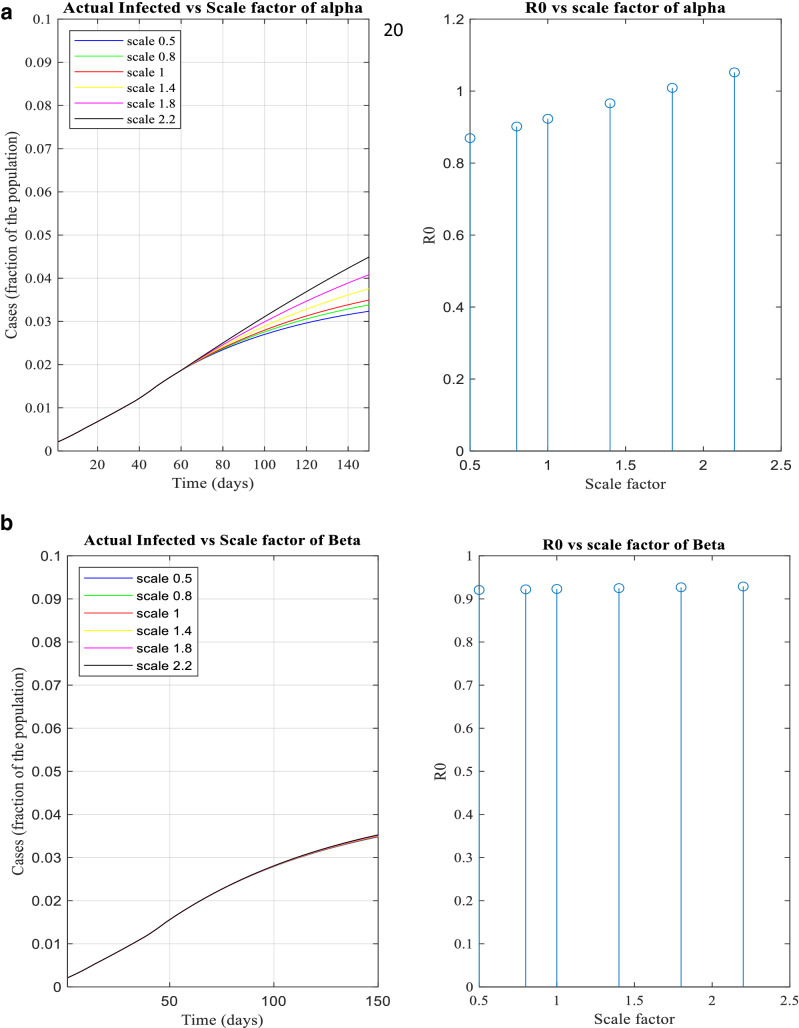

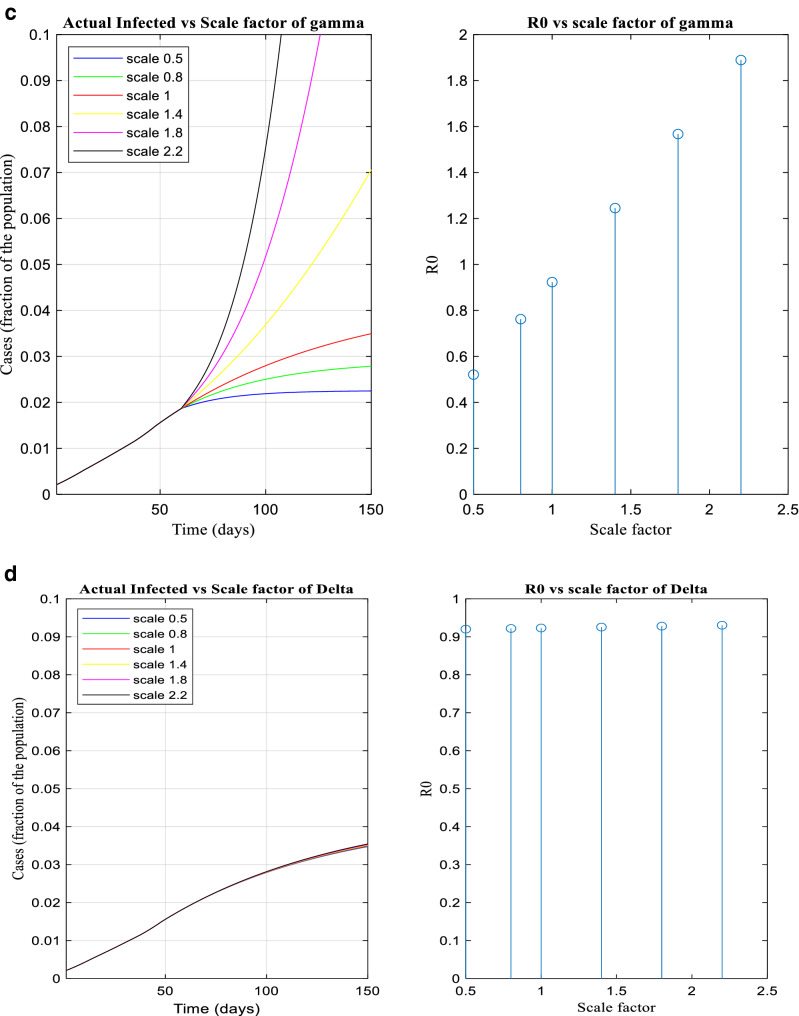
(**a**-**d**) The infected and R_0_ versus scale factor of α, β, γ and δ.

Figure 8 depicts ‘how infected case changes with respect to transmitting parameters. Figure 8a-d illustrates how the nature of pandemic changes along with R_0_ based on the transmitting parameters, α, β, γ, δ. The parameters are scaled using scaling factors 0.5, 0.8, 1.4, 1.8 and 2,2 on their current value during period 5.

**Figure 9:**
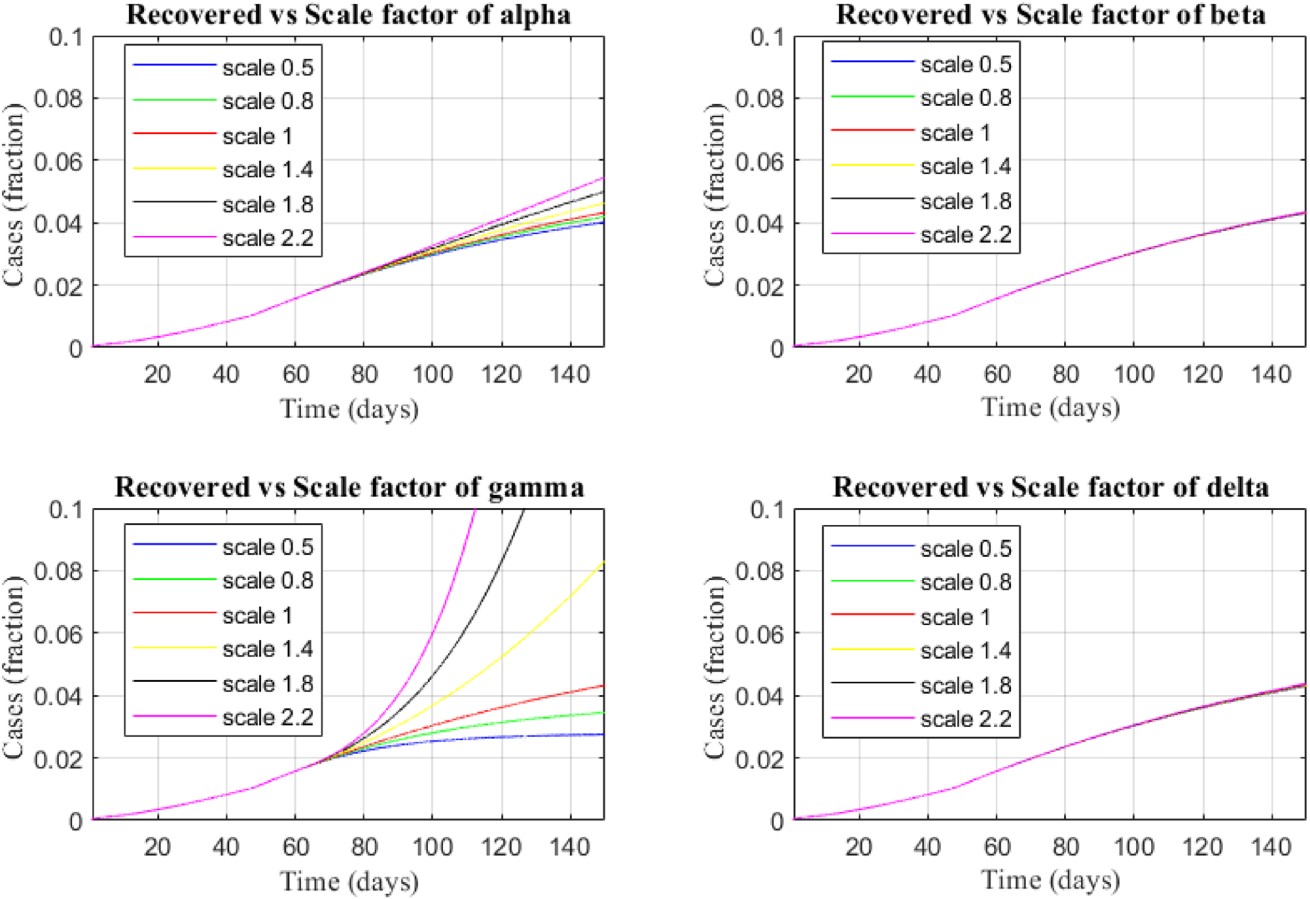
The sensitivity of transmission parameters over recovered case.

Figure 9 shows the sensitivity of transmission parameters with respect to the recovered cases.

**Figure 10:**
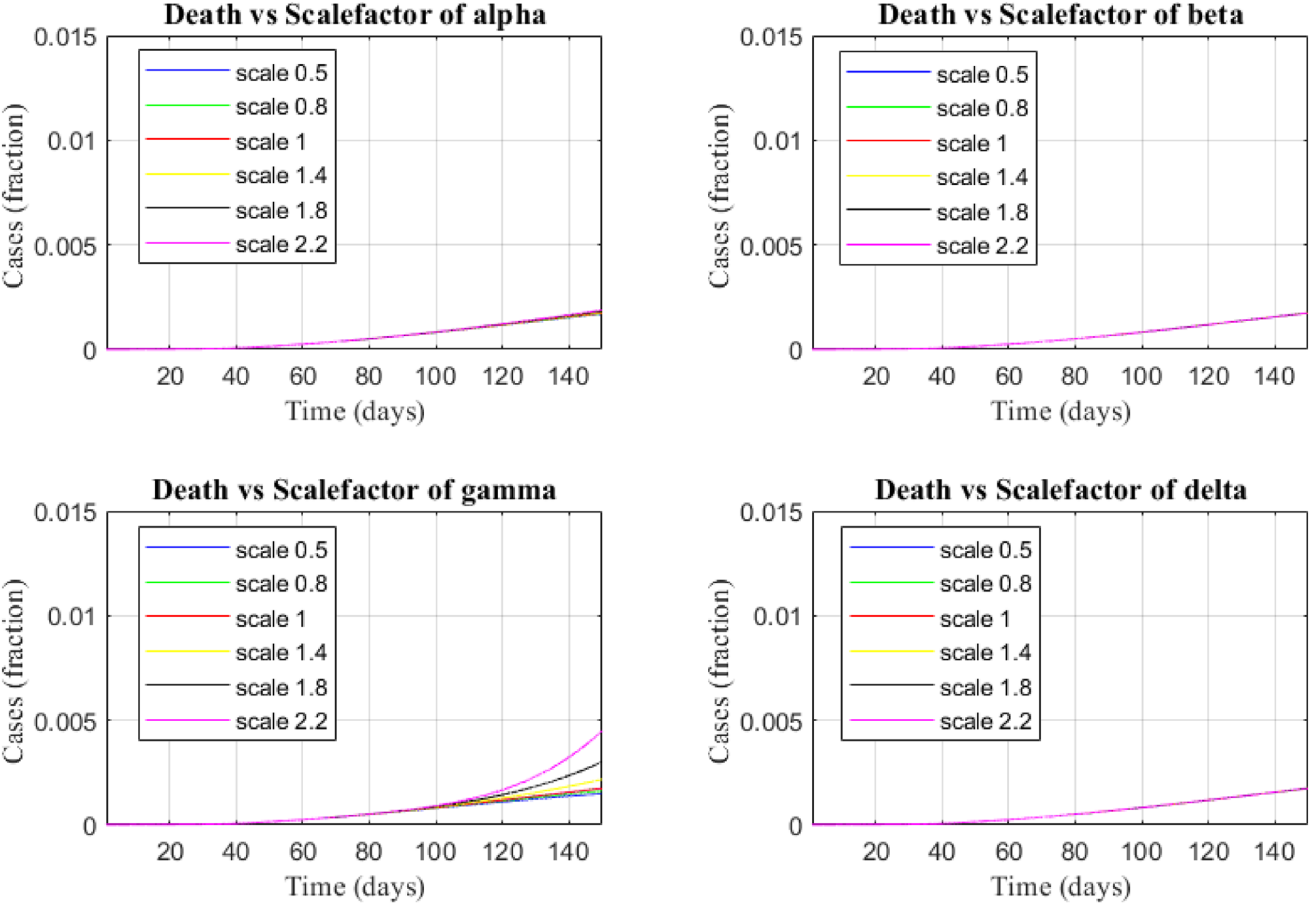
The sensitivity of transmission parameters over death cases.

Figure 10 shows the sensitivity of transmission parameters with respect to the death cases.

## Discussion

Based on the public data^38,39^, the situation in Oman at the end of May was not very severe. Muscat governorate reported more cases compared to other governorates. From the beginning itself, the Oman government has imposed strict measurements to control the disease. Oman Supreme committee has taken a good decision to close the main containment zone, Muscat governorate by April 10 which resulted in the reduction of the transmission rate. By April 29, 2020, the government lifted the restriction on travel inside the governorate except in and out of the Muscat governorate. More number of random tests were conducted in the main containment zones of Muscat governorate such as Muttrah, Seeb, Muscat Province and Al Amerat. It was made compulsory to wear masks in public places and thermal imaging was also done while entering majority of shops in Muscat. The entry to shops was restricted to people with a temperature of more than 37.5 °C, and children of age less than 12 years of old were also not permitted. These decisions were a part of several measures broadcasted by the Municipality as per decision number 199/2020, which lists out 15 safety measures to be practiced by the commercial establishments. Fines were also imposed on those who violates the rule. Lockdown in Muscat province, including the capital Muscat, was lifted entirely on Friday, May 29, as the government began easing the containment measures in place, nationwide. Additionally, at the end of May 2020, the government announced that at least 50 percent of public sector employees could resume working from their offices from Sunday, May 31.

In this research, the significant 15 parameters mentioned in the proposed model is calculated during the said period (Period 1–Period 5) mentioned in Sect. 3, where significant change is observed in the data. The corresponding basic reproduction number R_0_ is calculated and this depicts the nature of the pandemic with respect to the the spread of the disease.

During Period 1, a partial lifting of the restrictions was observed after a 2-month lockdown period. The value of R_0_ is found to be 1.5802. During Period 2, the ministry ensured strict social measures to stop the spreading of the disease. Timely instruction is given through official social media account^39^, such as the importance of washing hands, social distancing etc. At the same time, authorities took necessary care to convey the people not to be panic so that they can reduce the overstress in this situation. Otherwise, ultimately it will affect the living conditions and the business and working environments. More tests were conducted to avoid panic in people. As a result, a slight change resulted in social parameters like $\alpha$, $\beta$ and the new reproduction number becomes R_0_ = 1.4811.

During Period 3, isolation and quarantine policies were made stricter during the period from 28th June-10th July 2020. Anyone who enters the Sultanate was subjected to quarantine for 14 days. The Omani residents were not permitted to go outside the Sultanate. The land and air borders were closed. Because of this strict isolation and quarantine process, and basic social distancing measures among people (such as washing hands often, not touching one’s face, avoiding handshakes etc.) the value of R_0_ became 1.3071. During the period 11th July–20th July 2020, due to the lack of strict lockdown and an increase in the number of random tests in the containment zones, more infected cases were reported. The Supreme Committee in Oman advised the public to wear face masks in public places, and for business establishments to ensure that the visitors also followed the required safety measures. The spread of the disease was little more during this period, which resulted in R_0_ value of 1.4392.

From 21st July–30th July, a nationwide lockdown came into effect. Travel between the governorates was not allowed. The emergency services such as ambulances, service vehicles that provide electricity and water maintenance, and vehicles belonging to the police and Armed Forces, were exempted from this ban. People remained indoors between 7 P.M. and 6 A.M. It was not permitted at all to go outside after 7 P.M., either on foot or in a vehicle. Such incidents were imposed with proper fines. Royal Oman Police set several checkpoints on the roads that connect diverse areas of the Sultanate, to ensure domestic travel restrictions. Working was no longer a valid reason for going out of the house; progressively non-indispensable activities were also stopped. As a result, at the end of July, R_0_ value became 0.9761. It shows that the restriction of the contact with each other in various form significantly brings down the value of R_0_, thereby decreasing the spread of the disease.

Based on the parameters obtained during period 5 (21st July–30th July 2020) and with the assumption that the policy will remain the same, we predicted the infected size, recovery size and death cases up to 150 days. It is expected to have the number of cumulative infected cases as 236,436, the number of recovery cases as 207,839 and no of deaths as 9234 at the end of 150 days starting from June 04, 2020, provided no change in the policy decisions as on during period 5.

The sensitivity of the model is also analyzed on infected, recovered and death cases concerning transmission parameters as given in “Results” section. The sensitivity analysis on infected case shows that the transmission parameters alpha and gamma are very much sensitive to the spread of the disease. The reason for the high sensitivity of gamma is due to the fact that Moderate category people (M) have a higher probability rate of transmitting the disease as they are in the infectious stage and they are only isolated to their homes and, not admitted in the hospital. The people in the exposed (E) category have a higher rate of a chance of contact with other people as at this stage disease is not diagnosed, and it is infectious to a certain extent which results in higher sensitivity of alpha parameter. The lockdown and social distancing measures such as wearing a mask, avoiding gathering, closing schools and mosques etc. will help significantly reducing the value of these parameters.

The sensitivity analysis on recovered case shows that as the value of transmission parameters increases the number of recovered cases also increases. This is because the infected cases increase with the rise in parameters, and accordingly the number of recovered cases also rise due to the dedicated service of our health centers. In sensitivity analysis of death cases, as parameter value increases, the number of death case may also increase. But due to our medical service, the rise in the value may not be alarming. So, in addition to fineness in our medical field, it is a vital role to frame an appropriate policy to reduce the value of the parameter to control the disease like COVID-19 due to the absence of a specific vaccine for the disease.

It is observed that the transmission parameters are very much influenced by various remedial measures such as lockdown, social distancing and quarantine. This would reduce/increase the basic reproduction number which is the probability of disease transmission in a single contact multiplied by the average number of contacts per person. Since the transmission rate α is due to the contacts between susceptible and exposed (Et), its rate can be reduced through contact tracing and isolate them from the contact of others. The mass random test will also help to identify this category of exposed population (Et) who transmits the disease without showing any symptoms.

## Conclusion and future

In this work, we have developed a mathematical model to analyze the nature of the pandemic COVID-19 meaningfully on the data of the Sultanate of Oman. The model is an extension of SEIR where we expanded the infected compartments into mild (A), moderate (M), severe (H) and critical (C), based on the clinical stages of infection. The exposed category is also extended to E_N_ and E_T_ where E_T_ is an infectious stage. The parameters are estimated by fitting the data of Oman on the differential equations, and R_0_ is computed. The proof for the computation of R_0_, steady-state analysis and final pandemic size equations are also shown to validate the model. The parameter value is justified with the effective actions taken by the government to lessen the spread of the disease. The sensitivity of the transmitting parameters is also identified which is a direct indicator of the impact of the strict or weak lockdown measures on the spread of the disease. It is observed that the transmitting parameters alpha and gamma are sensitive to the number of infected cases, and their value can be reduced by proper contact tracing mechanism and effective social distancing measures. In conclusion in Oman, there is no need for any complete lockdown as per the present situation. This research can be extended by analyzing the data of Oman by taking Wilayat wise or governorate wise data in Oman. This can also be done for any country by considering the different factors such as population, culture, age distribution, regulations etc. Also, the model can be extended by incorporating the impact of suitable vaccination once it is discovered.
